# Supplementary material for: Moral Injury Among Medical Personnel and First Responders Across Different Healthcare and Emergency Response Settings: A Narrative Review
Source: Int J Environ Res Public Health. 2025 Jun 30;22(7):1055. doi: 10.3390/ijerph22071055 (PMC12294130; doi:10.3390/ijerph22071055)
Supplement: Supplementary file 1 [file ijerph-22-01055-s001.zip › ijerph-3658311-supplementary.pdf]

International Journal of
Environmental Research and Public Health

MDPI

Supplemental data Rimon et al.

Supplementary Table S1. Search strategy.

This table summarizes the search strategy used for the narrative review. It details the databases searched, the timeframe covered, the main search terms and keywords, and the inclusion and exclusion criteria applied during study selection.

DATABASE SEARCHED	TIMEFRAME	SEARCH TERMS/KEYWORDS USED	INCLUSION CRITERIA	EXCLUSION CRITERIA
**PUBMED**<br>**CENTRAL**<br>**GOOGLE SCHOLAR**<br>**SEMANTIC SCHOLAR**	2010–2025	“moral injury”, “moral distress”, “healthcare workers”, “first responders”, “medical personnel”, “PTSD”	Peer-reviewed studies on moral injury among healthcare workers or first responders; original data; any healthcare/emergency setting	Studies without original data; military-only populations; studies focused solely on burnout/occupational stress

Supplementary Table S2. Manuscripts described in this review. detailing their serial number, study reference, study design, population type, study setting, and primary focus. Each entry provides a concise overview of the reviewed studies, including cross-sectional and longitudinal designs, the populations studied (such as healthcare workers and public safety personnel), geographic locations (e.g., Pakistan, United States, Canada, multiple countries), and the main research themes addressed, such as prevalence, risk factors, and impacts related to mental health outcomes.

Serial No.	Study	Study Design	Population Type	Setting	Primary Focus	During Covid 19 Pandemic?
1.	Akhtar et al., 2022[10]	Cross-sectional	Healthcare workers	Pakistan	Prevalence, risk factors	V
2.	Amsalem et al., 2021[11]	Longitudinal	Healthcare workers	United States	Prevalence, impacts	V
3.	Campbell et al., 2024[9]	Cross-sectional	Healthcare workers	United States	Impacts	X

4.	Coimbra et al., 2024[33]	Cross-sectional meta-analysis	Healthcare workers	Multiple countries	Prevalence, impacts	V
5.	D'alessandro-Lowe et al., 2024[34]	Cross-sectional	Public safety personnel	Canada	Impacts	X
6.	Dale et al., 2021[17]	Longitudinal	Healthcare providers	United States	Prevalence, impacts	V
7.	Fatima et al., 2023[37]	Cross-sectional	Physicians	Pakistan	Prevalence, risk factors, impacts	V
8.	Gilbert-Ouimet et al., 2022[31]	Cross-sectional, mixed methods	Healthcare workers	No mention found	Risk factors	V
9.	Hegarty et al., 2022[32]	Cross-sectional qualitative	Healthcare workers	England	Impacts	V
10.	Hines et al., 2021[29]	Longitudinal	Healthcare workers	No mention found	Prevalence, risk factors	V
11.	Laher et al., 2022[27]	Systematic review	Nursing and care home staff	No mention found	Prevalence, risk factors, impacts	V
12.	Lennon et al., 2023a[36]	Cross-sectional	Healthcare workers	United States	Prevalence, impacts	V
13.	Lentz et al., 2021[2]	Scoping review	Firefighters, paramedics, police officers	No mention found	Risk factors, impacts	X
14.	Litam and Balkin, 2020[19]	Cross-sectional	Healthcare workers	No mention found	Impacts	V
15.	Mantri et al., 2020a[7]	Cross-sectional	Healthcare professionals	United States	Prevalence, risk factors	X
16.	Mantri et al., 2020b[20]	Cross-sectional	Healthcare professionals	United States	Prevalence, impacts	X
17.	Mooren et al., 2024[35]	Cross-sectional	Police officers	Netherlands	Impacts	X
18.	Nieuwsma et al., 2022[12]	Cross-sectional	Healthcare workers, veterans	United States	Prevalence, impacts	V
19.	Norman et al., 2023[22]	Cross-sectional with longitudinal	Veterans, healthcare workers, first responders	United States	Prevalence, impacts	X
20.	Papa et al., 2024[23]	Cross-sectional	Healthcare providers	United States	Prevalence, impacts	V
21.	Purcell et al., 2024[40]	Cross-sectional mixed-methods	Healthcare workers	United States	Prevalence, risk factors, impacts	V

22.	Rushton et al., 2021 [5]	Cross-sectional	Healthcare workers (nurses subgroup)	U.S. mid-Atlantic region	MI, resilience, prevalence	X
23.	Arielle et al., 2024[14]	Cross-sectional mixed-methods	Healthcare workers	United States, Canada	Risk factors, impacts	V
24.	Mady et al., 2020[28]	Critical review	Nurses	No mention found	Impacts	V
25.	Wang et al., 2021a[6]	Cross-sectional	Healthcare professionals	China	Prevalence, impacts	X
26.	Wang et al., 2021b[18]	Cross-sectional	Healthcare professionals	China	Prevalence, impacts	V
27.	Weber et al., 2022[38]	Cross-sectional and longitudinal	Healthcare workers	United States	Prevalence, impacts	X
28.	Zahiriharsini et al., 2022[39]	Cross-sectional	Healthcare workers and leaders	Canada	Risk factors	V
29.	Zerach and Levi-Belz, 2021[30]	Cross-sectional	Health and social care workers	Israel	Impacts	V
30.	Zerach and Levi-Belz, 2022[16]	Cross-sectional	Health and social care workers	Israel	Impacts	X
31.	Tao et al., 2024[24]	Cross-sectional	Acute care nurses	United States	Scale validation, MI, depression, anxiety, burnout	V
32.	Fino et al., 2023[25]	Cross-sectional	Healthcare workers (nurses, doctors, others)	Italy	Scale validation, depression, anxiety, PTSD, burnout	X
33.	Čartolovni et al., 2021[8]	Scoping review	Healthcare professionals	Healthcar e	MI concept	X
34.	Liu et al., 2023[26]	Cross-sectional	Healthcare professionals	China	Burnout, suicidal ideation, MI	X
35.	Kreh et al., 2021[42]	Scoping review	Healthcare workers	Not specified	Moral distress, MI, COVID-19	V
36.	Mantri et al., 2021[21]	Scoping review	Healthcare professionals	Not specified	MI, COVID-19, mental health	V
37.	Griffin et al., 2021[43]	Rapid review	Healthcare workers	Not specified	MI, COVID-19, rapid review	V
38.	Daphna-Tekoah et al., 2025[41]	Qualitative	Bereaved families	Israel	Moral trauma	X
39	Brennan et al., 2024[4]	Systematic review and	Multiple occupational groups worldwide	Various work	Prevalence of moral injury/PTED	X

		meta-analysis		environments globally		
40	Maguen et al. 2025[13]	Meta-analysis	Veterans, HCWs, First Responders	International	Moral injury rates	X
41	Hubbell et al. 2025[15]	Cross-sectional survey quantitative study	Registered nurses, United States	Hospital inpatient care settings	Resilience, moral injury, burnout	X
